# Anti-Wrinkle Effect of *Isatis indigotica* Leaf Extract: Evaluation of Antioxidant, Anti-Inflammation, and Clinical Activity

**DOI:** 10.3390/antiox10091339

**Published:** 2021-08-25

**Authors:** Jin Hyeok Kim, Dan Gao, Won Seok Jeong, Cheong Taek Kim, Chong Woon Cho, Hyung Min Kim, Jong Seong Kang

**Affiliations:** 1 College of Pharmacy, Chungnam National University, Daejeon 34134, Korea; oojh52@cnu.ac.kr (J.H.K.); gaodan@o.cnu.ac.kr (D.G.); 2 RNS Inc., Daejeon 34014, Korea; zmal1329@biorns.com (W.S.J.); happilion@biorns.com (C.T.K.)

**Keywords:** antioxidant, anti-inflammatory, anti-wrinkle, *Isatis indigotica* leaf, clinical study

## Abstract

*Isatis indigotica* leaf is an oriental herbal medicine that has been known for various pharmacological effects. However, its anti-wrinkle activity has not been fully evaluated. Therefore, we evaluated the anti-wrinkle effect of *I*. *indigotica* leaf extract on human skin. The purified extract inhibited 85.4% of 2,2-diphenyl-1-1picrylhydrazyl and 72.2% of 2,2′-azino-bis(3-ethylbenzothiazoline-6-sulfonic acid) diammonium salt radicals at a concentration of 1 mg/mL. Nitrite production was reduced by 30% after treatment with 50 μg/mL of extract. Three fractions from the extract downregulated the mRNA expression of matrix metalloproteinase-1 and -3 and upregulated the expression of interleukin 4. Among the three fractions, fraction 2 exhibited the highest activity. The major component of the extract was identified as 3,4,5-trimethoxycinnamic acid by liquid chromatography coupled with mass spectrometry. Molecular docking was conducted to predict the binding mechanism of 3,4,5-trimethoxycinnamic with matrix metalloproteinase-1 and -3, and their binding energies were −5.20 and −4.89 kcal/mol, respectively. In a clinical trial, five roughness values of visiometer and visual score were significantly reduced in treated groups compared with the placebo group after 8 weeks. *I. indigotica* leaf extract inhibits wrinkle formation, and could be a potential anti-wrinkle agent. This is the first clinical trial demonstrating its anti-wrinkle activity.

## 1. Introduction

Wrinkles, a major symptom of skin aging, are caused by multiple factors [1]. Reactive oxygen species (ROS) are one of the important factors responsible for wrinkle formation [2]. Oxidative stress induced by ROS promotes the production of the pro-inflammatory tumor necrosis factor (TNF)-α and interleukins (ILs). The pro-inflammatory mediators in the skin cause matrix metalloproteinase (MMP) activation [3]. MMPs degrade the matrix, causing the accumulation of degraded matrix components and unexpected cytokines. These lead to the loss of skin elasticity and moisture and, ultimately, wrinkle formation [4]. Therefore, anti-wrinkle agents must display antioxidant and anti-inflammatory activity, and must down-regulate the mRNA expression of MMPs and ILs.

For decades, efforts to develop anti-wrinkle products using herbal medicines have steadily increased [5]. For example, Park et al. reported that *Acanthopanax senticosus* extract could protect skin from wrinkle formation caused by the collagen synthesis of fibroblast cells and photo-irradiation by ultraviolet B light in hairless mice [6]. Ko et al. have demonstrated that sulforaphane, which is found in cruciferous vegetables, relieves premature skin aging [7]. However, these extracts and compounds are either very difficult to obtain or relatively expensive. Therefore, it is imperative to find a ubiquitous and inexpensive natural extract that can be used as an active ingredient.

The dry leaf of *Isatis indigotica* Fort is a widely used, low-cost herbal medicine known for its antioxidant and anti-inflammatory activity [8]. Our previous study reported excellent anti-wrinkle potential from *I. indigotica* leaf through the inhibition of MMP-1, MMP-3, and pro-inflammatory cytokine IL-β mRNA expression [9]. To elucidate the anti-wrinkle mechanism of the enriched *I. indigotica* leaf extract, we separated the enriched *I. indigotica* leaf extract into three fractions by column chromatography and evaluated their anti-inflammatory, antioxidant, and anti-wrinkle activities. We also evaluated the clinical effect and safety of an anti-wrinkle cream product formulated from enriched *I. indigotica* leaf extract and compared the activity between the placebo and treated groups.

## 2. Materials and Methods

### 2.1. Herbal Sample and Reagents

*I. indigotica* leaf was purchased from an online market (http://hanyakjae.net/, accessed on 5 January 2020) and produced in July 2019 from China. Potassium persulfate, 3,4,5-trimethoxycinnamic acid (TMCA, CAS 90-50-6), 2,2-diphenyl-1-1picrylhydrazyl (DPPH, CAS 1898-66-4), and 2,2′-azino-bis(3-ethylbenzothiazoline-6-sulfonic acid) diammonium salt (ABTS, CAS 30931-67-0) were purchased from Alfa Aesar (Haverhill, MA, USA). Distilled water was freshly produced using a Milli-Q system (Shinhan Company, Seoul, Korea). All reagents used in this study were of analytical grade. Acetonitrile, methanol, ethyl acetate, ethanol, 1,3-butylene glycol, and dimethyl sulfoxide (DMSO) were purchased from Burdick & Jackson (Muskegon, MI, USA). The primers were obtained from Bioneer Inc. (Daejeon, Korea). The absorbance values for antioxidant activity assays were measured using an infinite F200 microplate reader from Tecan (Mannedorf, Switzerland).

### 2.2. Preparation of I. indigotica Leaf Extract and Its Fractions

Cyclohexane (2.5 L) was added to 500 g of homogenized *I. indigotica* leaf to remove the lipophilic components. It was then collected through filter paper and extracted with 2.5 L of methanol at room temperature for 72 h. It was filtered again, and the filtrate was completely dried. Then, 5 volumes of ethyl acetate were added to the methanol extract and mixed for 24 h. The ethyl acetate solution was filtered, and 5 volumes of methanol were added to the filtrate. The mixture was completely dried by vacuum concentration. *I. indigotica* leaf extract was fractionized by silica gel (1.0 × 80 cm) column chromatography and was sequentially eluted with ethyl acetate–methanol (9:1, 4:1, and 2:1) to obtain three fractions.

### 2.3. Cell Culture

Mouse macrophage cell lines (RAW 264.7) and CCD-986sk human fibroblast cells were purchased from the Korean Cell Line Bank (Seoul, Korea). All cell lines were cultured and maintained in Iscove’s Modified Dulbecco’s Medium (Hyclone Co., Logan, UT, USA) supplemented with 10% fetal bovine serum (Hyclone Co., Logan, UT, USA) and 1% antibiotic/antimycotic (100 U/mL penicillin and 100 μg/mL streptomycin) at 37 °C in a 5% CO_2_ incubator.

### 2.4. Antioxidant Potential of I. indigotica Leaf Extract

Five different concentrations of the sample were prepared to evaluate the scavenging property of DPPH^•^ [10]. *I. indigotica* leaf extract (0.1 mL) was added to 3.9 mL of a 6 × 10^−5^ mol/L DPPH^•^ solution and stored at room temperature for 30 min. The absorbance was measured at 515 nm. ABTS radical cations were produced by reacting 7 mmol/L ABTS solution with 2.45 mmol/L potassium persulfate and stored in the darkroom at room temperature for 16 h [11]. Then, 25 μL *I. indigotica* leaf extract was added to 250 μL of distilled ABTS^+•^ solution with ethanol. After 4 min, the absorbance was measured at 734 nm. Ascorbic acid was used as a positive control. The DPPH and ABTS radical scavenging activities (%) were calculated as follows: [(1-absorbance of sample/absorbance of negative control) × 100]. The half maximal inhibitory concentration (IC_50_) was calculated by plotting a curve after log transformation.

### 2.5. Anti-Inflammatory Potential of I. indigotica Leaf Extract

To evaluate cell toxicity, the EZ-Cytox Cell Viability Assay Kit from DoGenBio (Seoul, Korea) was used. Cells were cultured in 96-well plates; then, five different concentrations (1, 5, 10, 25, and 50 μg/mL) of *I. indigotica* leaf extract in DMSO were added. The absorbance was measured at 450 nm [12].

To determine the amount of nitrite produced by macrophages, RAW 264.7 cells (1  ×  105 cells/mL) were cultured in a 96-well plate for 2 h. An aliquot of 200 μg/mL *I. indigotica* leaf extract and dextran (10 μM) were added. Then, the cells were incubated with lipopolysaccharide (LPS) (1 μg/mL) at 37 °C for 18 h. The positive control was 500 μg/mL of aspirin. The mixture of Griess reagent and cell culture supernatants was measured at 550 nm [13].

### 2.6. Anti-Wrinkle Activity of I. indigotica Leaf Fractions

The quantitative real-time polymerase chain reaction (RT-PCR) was used to evaluate mRNA expression. CCD-986sk cells were added to a 6-well plate at a concentration of 5 × 10^4^ cells/well and incubated in a 5% CO_2_ incubator at 37 °C. Then, the three *I. indigotica* leaf fractions dissolved in DMSO (5 μg/mL) were added to the cells for 24 h. Following the manufacturer’s instructions, total RNA was isolated from the CCD-986sk cells using TRIzol reagent from Life Technologies (cat. no.: 15596018, Grand Island, NY, USA), and the RNA was stored at −70 °C. PCR was performed on a 7500 Real-Time PCR System from Applied Biosystems (Foster City, CA, USA) using SYBR^®^ Premix Ex TaqTM from Takara Bio Inc. (Shiga, Japan) to measure mRNA. Data were expressed as the ratio of target mRNA expression to 36B4 (vehicle) mRNA expression. The primers used in this study were the same as those previously described [9]. The experiments were performed in triplicate.

### 2.7. Phytochemical Profile Using LC-ESI-MS

A prominence UFLC system from Shimadzu (Kyoto, Japan) equipped with a CBM-20A Communication Bus Module, SPD-M20A Photodiode Array Detector, LC-20AD Pump, SIL-20A Autosampler, and CTO-20A Column Oven was used. After a 10 μL injection, the analytes were separated through a Hector-M C18 column (4.6 × 250 mm, 5 μm) from RStech (Daejeon, Korea) using mobile phase (A) 0.1% formic acid in water and (B) 0.1% formic acid in acetonitrile at a flow rate of 0.3 mL/min. The elution started at 0% B for 20 min and then was increased to 100% B for 110 min. The eluents were then detected by the LCMS-8040 system (Shimadzu) with an ESI interface source in positive and negative mode at 3.5 and −3.5 kV interface voltage, respectively. The MS source conditions were as follows: nebulizing gas flow rate, 3 L/min; desolvation line temperature, 250 °C; drying gas flow, 15 L/min; and heat block temperature, 400 °C.

### 2.8. Molecular Docking

AutoDock 4.26 was used for the molecular docking study to investigate the interaction of the main compound (TMCA) in *I. indigotica* leaf extract and creams with MMP-1 and MMP-3 [14]. The three-dimensional X-ray crystallographic structures of MMP-1(PDB ID: 966C) and MMP-3 (PDB ID: 1G4K) were downloaded from the Protein Data Bank. The structure of TMCA was constructed using Chemdraw 3D 18.0 software (Cambridge, Soft Co. Cambridge, MA, USA), and energy minimization using the force field MM2 approach was applied. The grid size was set with coordinates x = 126, y = 126, and z = 126 with spacing of 0.375 Å. Dockings were regularly ranked using AutoDock based on the lowest evaluated binding energies. The Discovery Studio 2020 Client program was used for graphic display.

### 2.9. Clinical Trials for Anti-Wrinkle Activity

The clinical study was conducted in accordance with the Korean Ministry of Food and Drug Safety (MFDS) guideline. The subjects included 23 females aged 37–59 years. All subjects spontaneously participated in the clinical trials and were in healthy condition, without acute or chronic disease but with wrinkles on the face.

An amount of 1 g of *I. indigotica* leaf extract was mixed with 99 mL of 1,3-butylene glycol, and then 2.5 g of a 1,3-butylene glycol solution including *I. indigotica* leaf extract was homogenized with the cream matrix [9]. The cream formulation with or without *I. indigotica* leaf extract was applied to the face skin twice a day, in the morning and evening, for 8 weeks. Anti-wrinkle activity was evaluated by two parameters: visual score and wrinkle quantification. The visual scores, which ranged from 0 to 9, were diagnosed by three dermatologists. A visual score of 0 meant none, whereas 9 represented severe. The wrinkles were quantified using a Skin-Visiometer SV600 from Courage + Khazaka electronic GmbH (Koln, Germany), and the visiometer roughness values R1 (skin roughness), R2 (maximum roughness), R3 (average roughness), R4 (smoothness depth), and R5 (arithmetic average roughness) were calculated, where a lower value referred to less-severe wrinkles [15]. This study was approved by the Institutional Review Board of the Korea Dermatology Research Institute (approval number: KDRI-IRB-19206) according to the requirements of the MFDS (Guidelines for Efficacy Evaluation of Functional Cosmetics, KFDA 11-1470000-000863-01, 2005, https://www.mfds.go.kr/brd/m_218/view.do?seq=708, accessed on 6 July 2020).

### 2.10. Statistical Analysis

Data were processed using Minitab (version 19.2) for statistical analysis. The significance between the experimental groups was evaluated using a paired Student’s *t*-test or analysis of variance (ANOVA) for results following a normal distribution when evaluated by the Ryan–Joiner normality test. Data that did not follow a normal distribution were validated by nonparametric statistical methods such as the Wilcoxon signed-rank test, Friedman test, Mann–Whitney U-test, or Kruskal–Wallis test. Data were presented as the mean and standard deviation. *p*-values less than 0.05 were considered statistically significant.

## 3. Results

### 3.1. Antioxidant Activity of I. indigotica Leaf Extract

**Figure 1:**
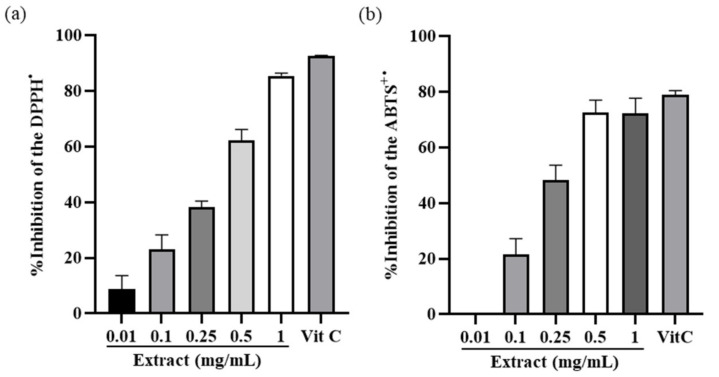
(**a**) DPPH and (**b**) ABTS radical scavenging activity of *I. indigotica* leaf extract at different concentrations. Vitamin C (ascorbic acid) was used as the positive control. Data were obtained through three independent replicates.

The DPPH radical scavenging activity was significantly increased in a concentration-dependent manner (Figure 1a). The highest DPPH radical scavenging activity (%) was observed with 1 mg/mL of *I. indigotica* leaf extract, which inhibited 85.4% of the DPPH radical. The ABTSradical scavenging activity was also increased in a concentration-dependent manner (Figure 1b). The maximum activity of the *I. indigotica* leaf extract was 72.6% at a concentration of 0.5 mg/mL. The IC_50_ in DPPH and ABTS assays were 0.46 mg/mL and 0.48 mg/mL respectively.

### 3.2. Anti-Inflammatory Activity of I. indigotica Leaf Extract

**Figure 2:**
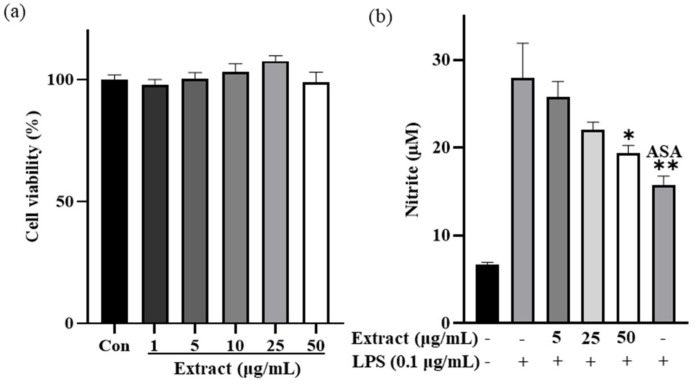
(**a**) Cell viability and (**b**) inhibitory activity of nitrite production in RAW 264.7 macrophages after treatment with different concentrations of *I. indigotica* leaf extract. RAW 264.7 macrophages were incubated with 0.1 μg/mL of lipopolysaccharide for 18 h. Acetylsalicylic acid (ASA) was used as the positive control. Data were obtained through three independent replicates. * and ** indicate significant differences at 0.05 and 0.01 significance level, respectively.

The cell viability of RAW 264.7 cells was determined after treatment with a range of concentrations of *I. indigotica* leaf extract. The viability of RAW 264.7 cells was not significantly affected by 50 μg/mL of extract (Figure 2a). The nitrite production showed a significant difference directly proportional to the concentration of *I. indigotica* leaf extract (Figure 2b). There was no inhibitory activity against nitrite production at 5 μg/mL of extract; however, nitrite production was greatly inhibited by treatment with 50 μg/mL of *I. indigotica* leaf extract. Moreover, there was no significant difference between 500 μg/mL of aspirin as a positive control and 50 μg/mL of *I. indigotica* leaf extract.

### 3.3. Anti-Wrinkle Activity of I. indigotica Leaf Fractions

**Figure 3:**
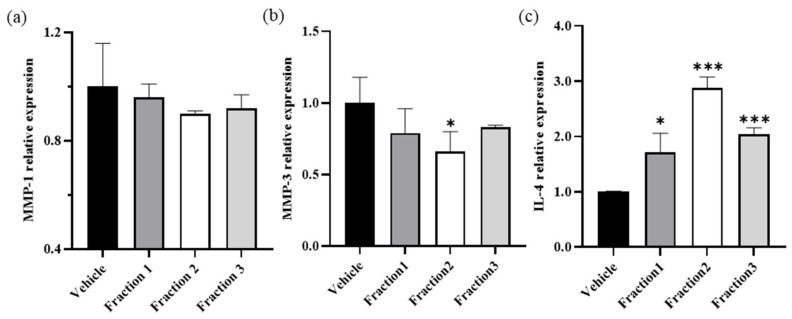
Effects on the mRNA expression levels of (**a**) MMP-1, (**b**) MMP-3, and (**c**) IL-4 in human fibroblast CCD-986sk cells following treatment with three *I. indigotica* leaf fractions. CCD-986sk cells were incubated in 5% CO_2_ at 37 °C, and then the three *I. indigotica* leaf fractions dissolved in dimethyl sulfoxide (5 μg/mL) were transferred into the cells for 24 h. The mRNA levels after treatment with the three fractions were assayed using real-time polymerase chain reaction. Data were obtained through three independent replicates. * and *** indicate significant differences at 0.05 and 0.001 significance level, respectively.

The possibility of anti-wrinkle property of *I. indigotica* leaf extract, including antioxidant and anti-inflammatory properties, was confirmed in this study. However, it is important to elucidate the active compounds responsible for these pharmacological effects. Therefore, *I. indigotica* leaf extract was fractionated for further evaluation. Then, the mRNA expression of MMP-1, MMP-3, and IL-4 was measured by RT-PCR in CCD-986sk cells after treating with three *I. indigotica* leaf fractions (Figure 3). The relative expressions of inflammatory cytokines MMP-1 (Figure 3a) and MMP-3 (Figure 3b) followed by treatment with *I. indigotica* leaf extract were lower compared with the vehicle-treated group. Meanwhile, the relative expression of IL-4 was higher in the group treated with *I. indigotica* leaf extract compared with the vehicle-treated group (Figure 3c).

### 3.4. Phytochemical Profile of I. indigotica Leaf Fractions

**Figure 4:**
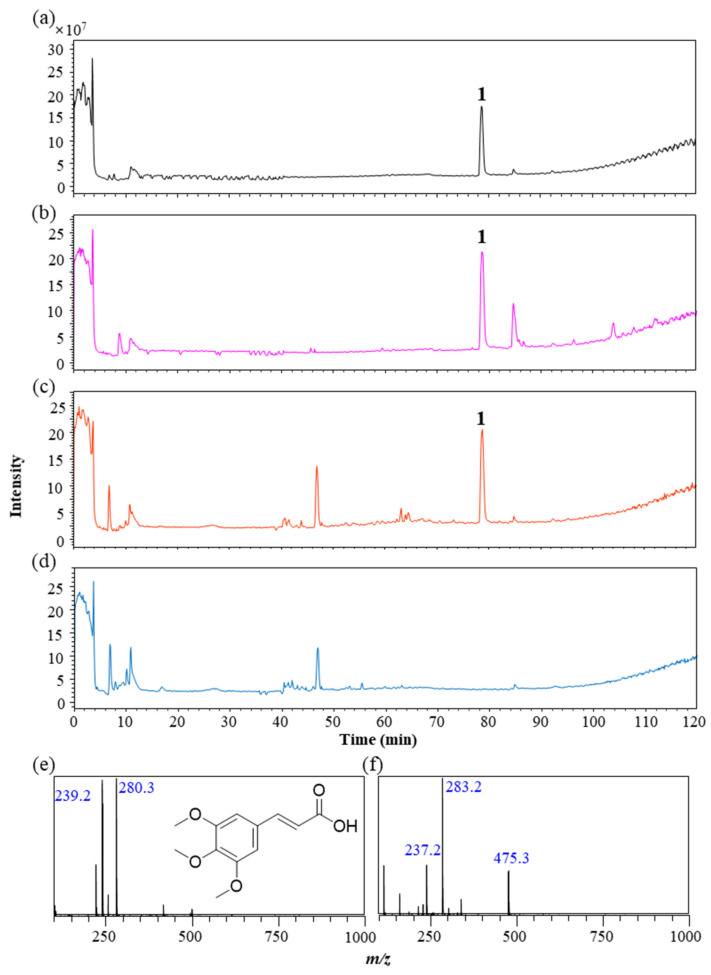
Identification of main compounds in three *I. indigotica* leaf fractions using LC-MS. Total ion chromatograms of (**a**) standard of 3,4,5-trimethoxycinnamic acid, (**b**) fraction 1, (**c**) fraction 2, and (**d**) fraction 3 at positive mode. MS spectra of peak 1 (3,4,5-trimethoxycinnamic acid) at (**e**) positive and (**f**) negative mode. LC conditions: column, Hector-M C18 column (4.6 mm × 250 mm, 5 m); flow rate, 0.3 mL/min; mobile phase, A-0.1% formic acid in water and B-0.1% formic acid in acetonitrile; elution program, 0% B for 20 min to 100% B 130 min; injection volume, 10 μL. MS conditions: interface, electrospray ionization; interface voltages, 3.5 kV at positive and −3.5 kV at negative; nebulizing gas flow rate, 3 L/min; desolvation line temperature, 250 °C; drying gas flow rate, 15 L/min; heat block temperature, 400 °C.

Based on the results of mRNA expression, fractions 2 and 3 exhibited higher anti-wrinkle activity. To identify the active compounds, three fractions were analyzed by liquid chromatography coupled with electrospray ionization–mass spectrometry (LC-ESI-MS), and the total ion chromatograms are shown in Figure 4. Fractions 1 and 2 contained the major compound at a retention time of 79 min, and fractions 2 and 3 also contained second highest compound at a retention time of 47 min. We focused on the main compound in fraction 2 (peak 1), since this fraction exhibited the highest anti-wrinkle activity. When analyzed by MS, peak 1 showed *m/z* 280.3 and *m/z* 239.2 in positive mode and *m/z* 283.2, *m/z* 237.2, and *m/z* 275.3 in negative mode, and their ion forms were predicted as [M+MeCN+H]^+^ and [M+H]^+^ in positive mode and [M+HCOO]^−^, [M–H+]^−^, and [2M–H+]^−^ in negative mode, respectively (Figure 4). The major compound in fraction 2 was identified as TMCAby comparing the retention time and MS spectra with the reference standard.

### 3.5. Molecular Docking

**Figure 5:**
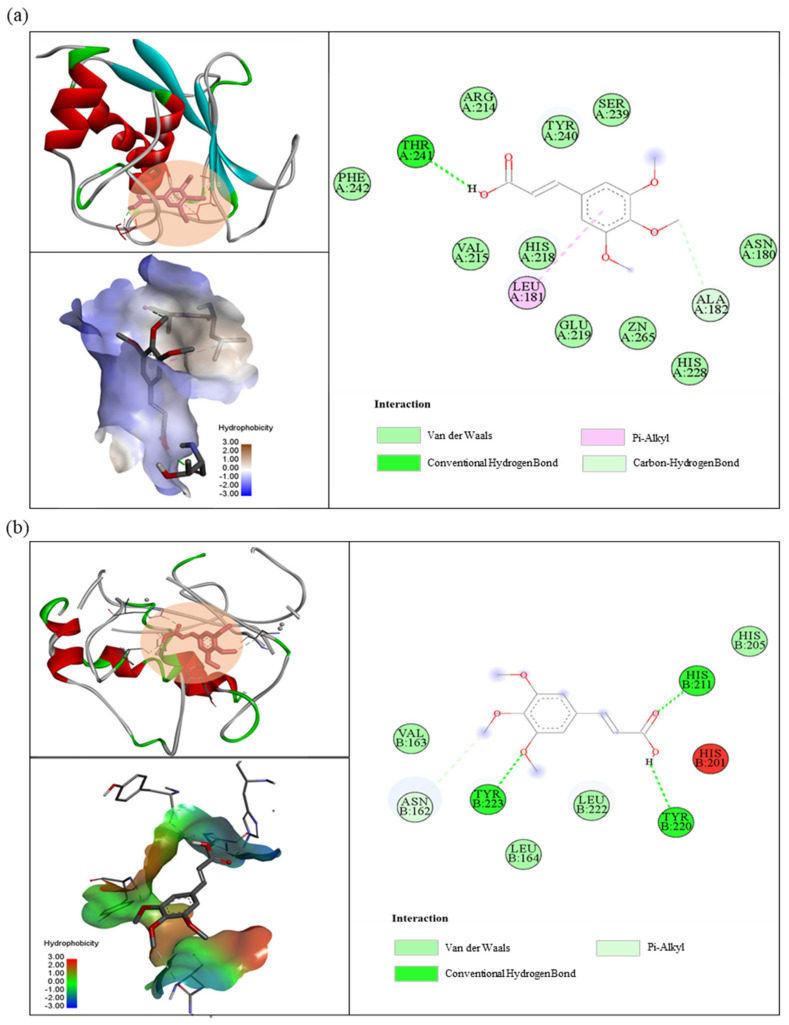
Molecular interaction of 3,4,5-trimethoxycinnamic acid with (**a**) MMP-1 and (**b**) MMP-3. The figure was built using PyMOL and Discovery Studio.

To predict the binding mechanism between TMCA and MMP-1 and MMP-3, docking simulations were performed using AutoDock tools. The docking simulation of TMCA with MMP-1 and MMP-3 revealed the best-returned position (Figure 5a,b) with a binding energy of −5.20 and −4.89 kcal/mol, respectively. Different types of interactions were identified between TMCA and the residues of the active pocket formed in MMP-1 and MMP-3, including pi-Alkyl, van der Waals interactions, conventional hydrogen bond, and carbon–hydrogen bond. For example, hydrogen bonding interactions between the active site of MMP-3 and TMCA involved hydrogen bonds in residues TYR 223, TYR 220, and HIS 211. The hydrophobic residues PHE 242, ARG 214, TYR 240, SER 239, VAL 215, HIS 218, GLU 219, ZN 265, HIS 228, and ASN 180 were observed to strengthen the interaction between TMCA and MMP-1 through van der Waals interactions.

### 3.6. Clinical Trial

**Figure 6:**
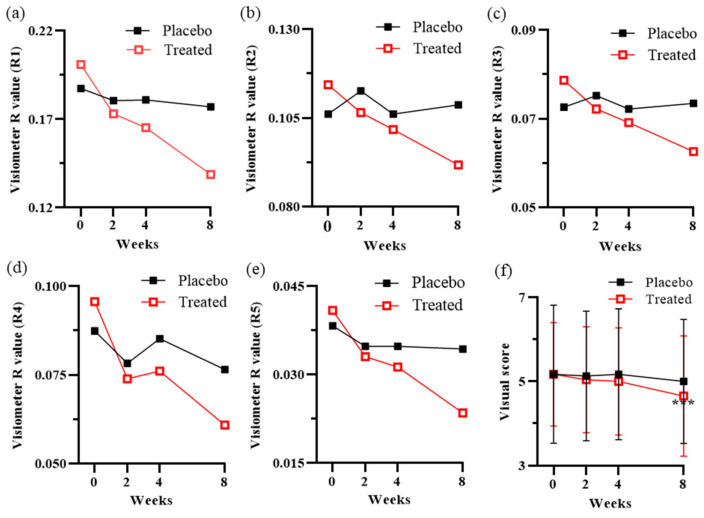
Differences of five visiometer R values quantified using Skin-Visiometer SV600 and visual score after treatment with a cream including *I. indigotica* leaf extract for 8 weeks between placebo and treated groups to evaluate anti-wrinkle activity. (**a**) R1: skin roughness. (**b**) R2: maximum roughness. (**c**) R3: average roughness. (**d**) R4: smoothness depth. (**e**) R5: arithmetic average roughness. (**f**) Visual scores graded by dermatologist were followed Gaussian distribution by the result of Ryan–Joiner normality test, and a significant difference *** of visual scores on the treated skin surface after 8 weeks treatment was proved by repeated-measures ANOVA at the 0.001 significance level.

**Figure 7:**
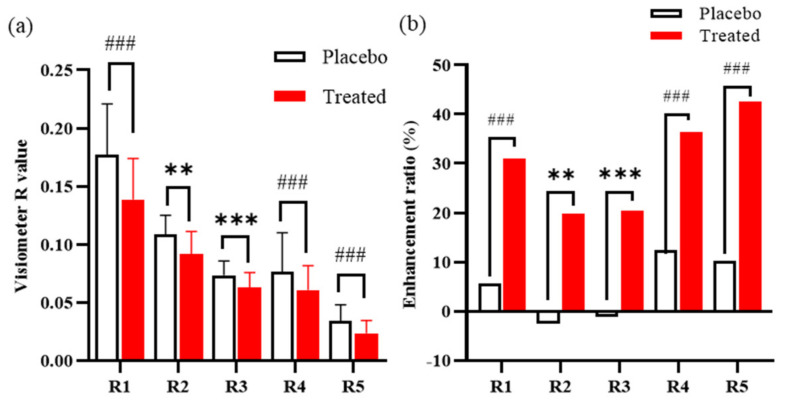
Comparison of (**a**) visiometer R values and (**b**) their enhancement ratio between placebo and treated groups after 8 weeks treatment with a cream including *I. indigotica* leaf extract. Enhancement ratios were calculated as the difference of R value between time 0 and 8 weeks divided by the R value at 8 weeks. ** and *** indicate significant differences at 0.01 and 0.001 significance level, respectively, by the results of repeated-measures analysis of variance when the normality was proved by Ryan–Joiner normality test. ^###^ indicates a significant difference at 0.001 significance level by the Friedman test when the normality was rejected by Ryan–Joiner normality test.

After 8 weeks of treatment with a cream containing *I. indigotica* leaf, subjects’ wrinkles were significantly reduced. Figure 6 shows the significant improvement in wrinkles after treatment for 8 weeks between the placebo and treated groups. The visiometer roughness values of R4 showed no difference at the 0.05 significance level between 2 and 4 weeks (Figure 6d). Except for R4, all visiometer roughness values were significantly (*p* < 0.05) and steadily reduced during the 8 weeks period. In addition, the visual score was reduced in the treatment group. This indicates that the wrinkles were decreased by treatment with *I. indigotica*-leaf-enriched cream (Figure 6f). After 8 weeks, all factors including visiometer roughness values and the visual score showed a significant difference compared with week 0 at the 0.01 significance level (Figure 7a). An enhancement ratio (%) was calculated as follows: |R8 week–R0 week|/R0 week × 100. The enhancement ratios between the placebo and treatment groups were compared (Figure 7b). The percentage of the enhancement ratio ranged from 20.5% in R3 to 42.6% in R5. These results indicate that *I. indigotica* leaf extract significantly reduced wrinkle formation.

## 4. Discussion

The study of the bioactivity and chemical constitution profile of natural products has increased as the development of drugs, functional cosmetics, and active ingredients using herbal medicines becomes more popular [16]. We evaluated the antioxidant, anti-inflammatory, and anti-wrinkle effects of *I. indigotica* leaf extract and related fractions. In addition, the molecular docking of the major compound was simulated to explain its mechanism of action. Lastly, the anti-wrinkle activity of the extract was evaluated in a clinical trial.

*I. indigotica* leaf extract exhibited DPPH and ABTS radical scavenging activity (Figure 1) and reduced nitrite production induced by LPS (Figure 2). The inhibition of radicals is important since excessive radicals cause damage to the epidermal barrier and induce wrinkle formation by decomposing collagen and elastin with concomitant protein oxidation and lipid peroxidation. In addition, alleviating oxidative stress may lead to increased anti-wrinkle activity since oxidative stress increases MMP-2 expression.

Excessive nitrite production causes skin tissue damage resulting from the inflammatory response. Nitrite may be transformed to peroxynitrite and induces inflammation. In our study, nitrite production was decreased by up to 70% when 50 μg/mL of *I. indigotica* leaf extract was administered, indicating that *I. indigotica* leaf extract can be used as an anti-wrinkle agent.

To identify its active component, *I. indigotica* leaf extract was separated into three fractions by column chromatography, and the fractions were used to measure the expression of MMP-1, MMP-3, and IL-4 mRNAs (Figure 3). Overexpression of MMP-1 and MMP-3 degrades proteoglycan, laminin, fibronectin, and non-fibrillar collagens, leading to the destruction of tissue and eventually to chronic inflammation. MMP-3 production is inhibited by IL-4 [17]. All three fractions inhibited the expression of MMP-1 (Figure 3a) and MMP-3 (Figure 3b), whereas the expression of IL-4 was enhanced (Figure 3c). In particular, fraction 2 showed the best results among the three fractions and increased IL-4 expression by 2.8-fold. However, this activity could have a harmful effect on patients with atopic dermatitis since the excessive expression of IL-4 induces allergic reactions [18].

The binding energy of TMCA with MMP-1 and MMP-3 was −5.20 and −4.89 kcal/mol, respectively. Therefore, TMCA is inhibited more by MMP-3 than by MMP-1 (Figure 5). This result corresponded with our mRNA expression experiments for MMP-1 and MMP-3 (Figure 3a,b) in which fractions 1 and 2 exhibited more inhibition of MMP-3 than MMP-1, whereas treatment with fraction 3 showed similar expression levels of MMP-1 and MMP-3. Rutin, a widely used anti-wrinkle agent from plants, showed a binding energy of −10.17 kcal/mol for MMP-3 [19,20]. Therefore, *I. indigotica* leaf extract could be used as a potential ingredient for anti-wrinkle activity, and TMCA may represent a suitable marker compound.

Finally, this study demonstrated a considerable enhancement in the treatment of wrinkles by *I. indigotica* leaf extract in a clinical trial. Five visiometer roughness values and visual score were reduced after 8 weeks of treatment (Figure 6), and the results were corroborated by parametric and nonparametric statistics (Figure 7). Among the five visiometer roughness values, R5 (arithmetic average roughness) showed the biggest difference, with an enhancement ratio of 32%.

## 5. Conclusions

This study demonstrated the anti-wrinkle activity of *I. indigotica* leaf extract. *I. indigotica* leaf extract showed DPPH and ABTS radical scavenging effects and inhibitory effects on nitrite production and MMP-1 and MMP-3 expression. Accordingly, *I. indigotica* leaf Folium extract may be useful as an anti-wrinkle agent, which was confirmed in a clinical trial for the first time. Furthermore, the main component was identified as a marker for quality control. Therefore, *I. indigotica* leaf extract may be used in commercial products to reduce wrinkle formation and improve skin aging.

## Data Availability

All data is contained within the article.

## Abbreviations

ROS	reactive oxygen species
TNF	tumor necrosis factor
IL	interleukin
MMP	matrix metalloproteinase
TMCA	3,4,5-trimethoxycinnamic acid
DPPH	2,2-diphenyl-1-1picrylhydrazyl
ABTS	2,2′-azino-bis(3-ethylbenzothiazoline-6-sulfonic acid) diammonium salt
IC_50_	half maximal inhibitory concentration
RT-PCR	real-time quantitative polymerase chain reaction
LC-ESI-MS	liquid chromatography coupled with electrospray ionization–mass spectrometry
LPS	lipopolysaccharide
MFDS	Korean Ministry of Food and Drug Safety
ANOVA	analysis of variance
